# Supplementary material for: Fueling sentinel node via reshaping cytotoxic T lymphocytes with a flex-patch for post-operative immuno-adjuvant therapy
Source: Nat Commun. 2023 May 2;14:2518. doi: 10.1038/s41467-023-38245-7 (PMC10154421; doi:10.1038/s41467-023-38245-7)
Supplement: Supplementary file 1 — Supplementary Information [file 41467_2023_38245_MOESM1_ESM.pdf]

Supplementary Information for

Fueling sentinel node via reshaping cytotoxic T lymphocytes with a flex-patch for post-operative immuno-adjuvant therapy

Bei Li[1,2,3], Guohao Wang[1,2,3], Kai Miao[1,2,3], Aiping Zhang[1,2], Liangyu Sun[1], Xinwang Yu[1,2], Josh Haipeng Lei[1,2], Lisi Xie[1,2], Jie Yan[1,2], Wenxi Li[1,2], Chu-Xia Deng[1,2]*, Yunlu Dai[1,2]*

[1]Cancer Centre and Institute of Translational Medicine, Faculty of Health Sciences, University of Macau, Macau SAR 999078, China.

[2]MoE Frontiers Science Center for Precision Oncology, University of Macau, Macau SAR 999078, China.

[3]These authors contributed equally.

*E-mail: yldai@um.edu.mo (Y. D.); cxdeng@um.edu.mo (C. D.)

Supplementary figures

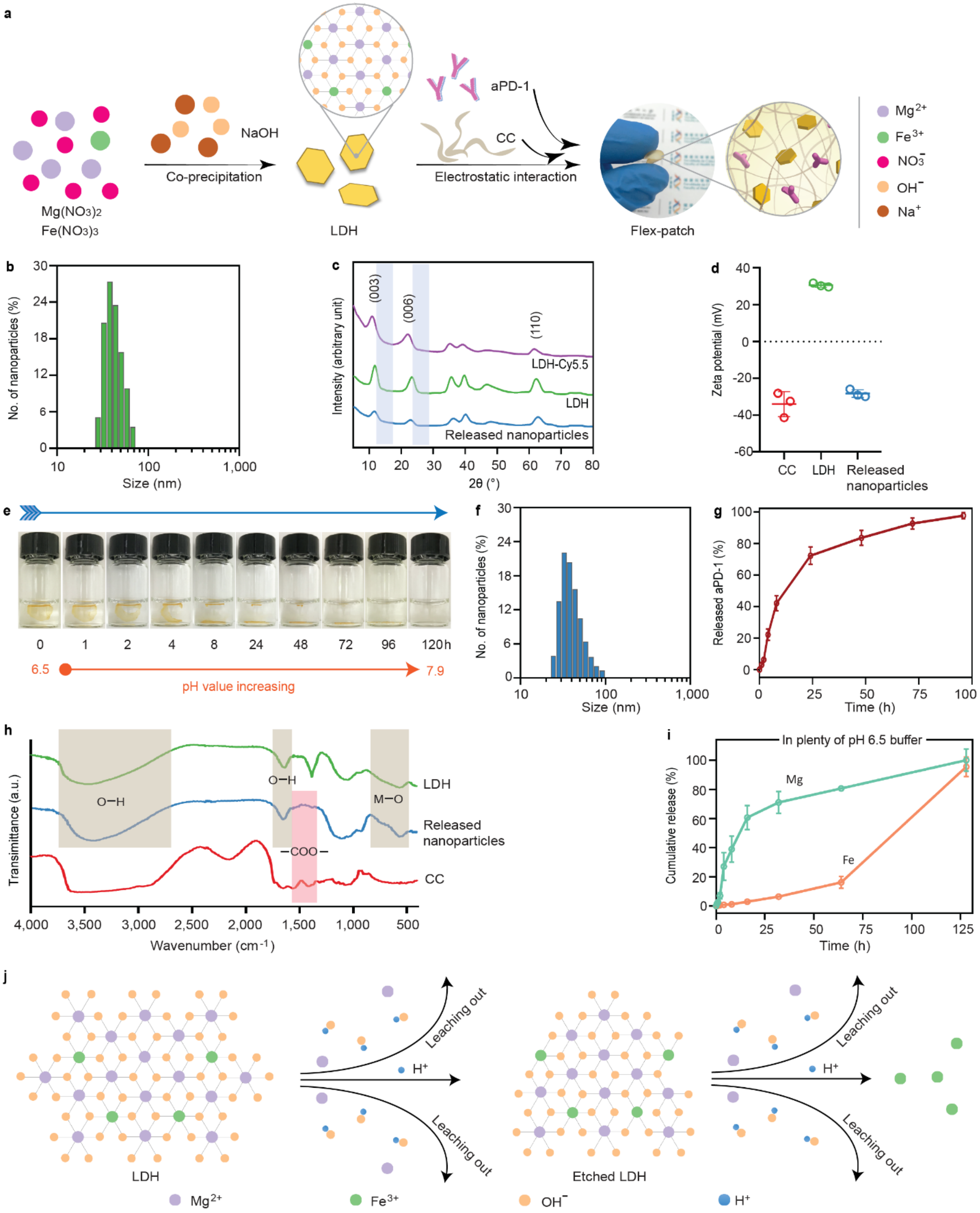

Supplementary Figure 1. Supplementary characterization of the flex-patch. a, The schematic illustration of the flex-patch design. **b**, Hydrodynamic size of LDH nanosheets determined by dynamic light scattering (DLS). **c**, XRD patterns of different samples. **d**, Zeta potentials of different samples. **e**, Representative photos to exhibit the in vitro disassembling of the patch in pH 6.5 phosphate buffer. **f**, Hydrodynamic size of disassembled nanoparticles determined by DLS. **g**, Cumulative release profile of aPD-1 from patch in pH 6.5 phosphate buffer. **h**, Fourier transform infrared spectroscopy (FTIR) of different samples. **i**, Cumulative etching of LDH in plenty of pH 6.5 phosphate buffer. **j**, Schematic illustrating the LDH degrading process. For (**d**,**g**,**i**), n = 3 per group; data are presented as the mean ± SD. For (**b**,**c**,**f**,**h**), experiments were performed three times independently with similar results. Source data are provided as a Source Data file.

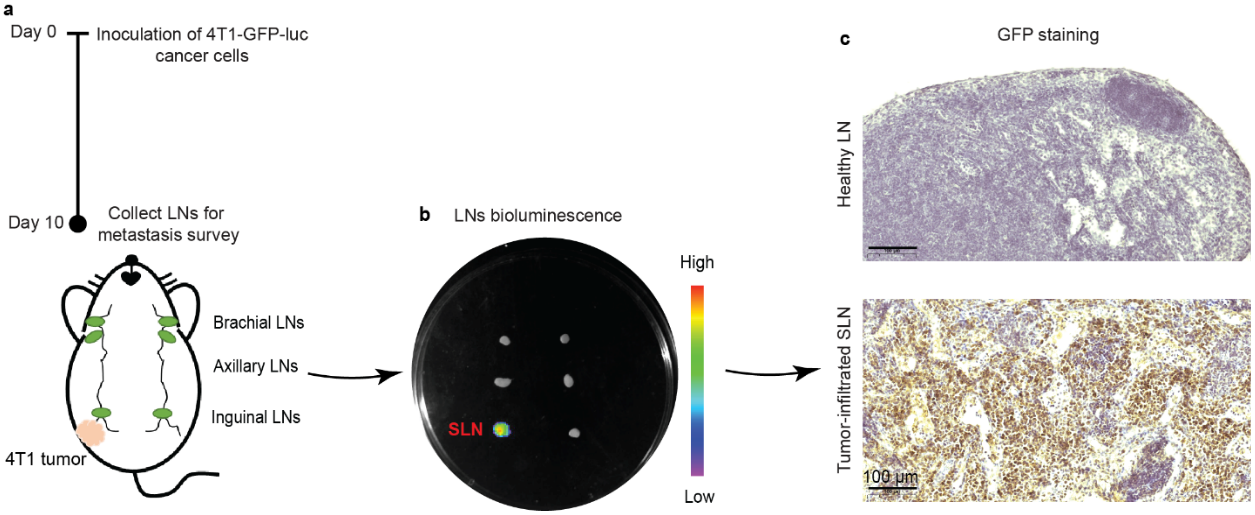

Supplementary Figure 2. SLN metastatic tracking in postsurgical 4T1 BC mouse model. a, Schematic illustrating the inoculation of 4T1-GFP-luc cancer cells to survey SLN metastasis. **b**, Ex vivo bioluminescence of LNs collected from 4T1-tumor model on Day 10 postinoculation. Here, LNs include two brachial LNs, two axillary LNs, and two inguinal LNs. The one next to the 4T1 primary tumor is regarded as SLN, which exhibited bioluminescence. **c**, IHC staining of GFP (4T1 marker) in healthy LN (up) and in metastatic SLN (down). Scale bar: 100 µm. n = 3 mice per group.

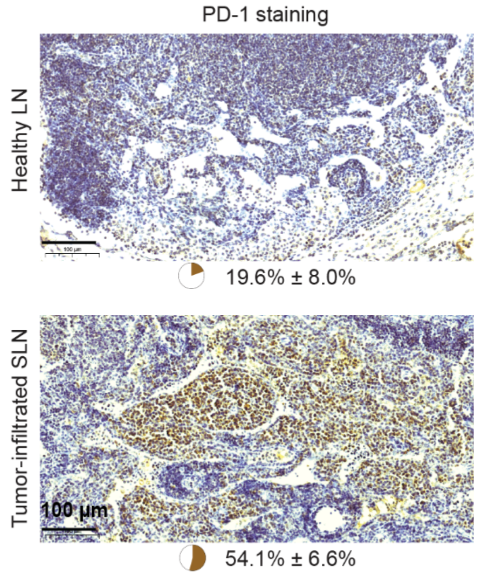

Supplementary Figure 3. PD-1 expression in the LN analyzed by IHC staining. PD-1 staining in healthy LNs (up) and in metastatic SLNs (down). For metastatic SLNs, IHC analysis was carried out on Day 10 postinoculation of the 4T1 BC mouse model. Scale bar: 100 µm. n = 3 mice per group. Data are presented as the mean ± SD. Source data are provided as a Source Data file.

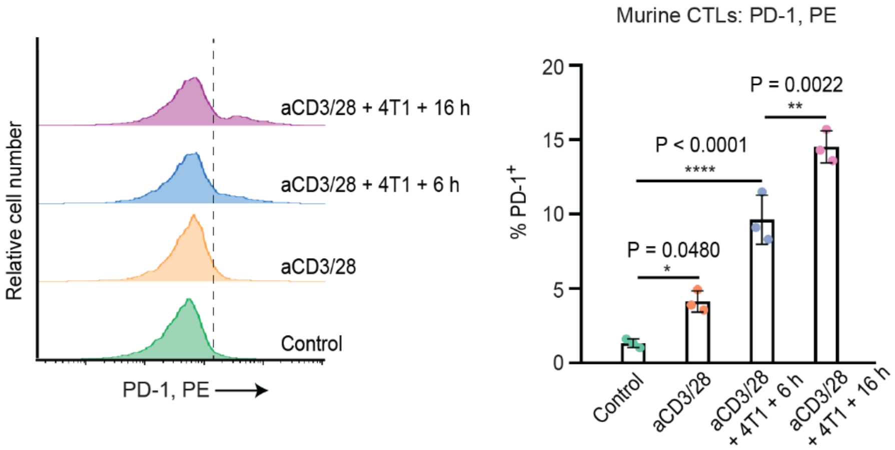

Supplementary Figure 4. PD-1 expression of murine CTLs stimulated by 4T1 cancer cells. Here, murine CTLs were pre-activated by aCD3/28. Prolonging the coculture of 4T1 cancer cells and murine CTLs could upregulate the surface PD-1 expression of CTLs. n = 3 per group. Data are presented as the mean ± SD, and statistical significance was calculated via one-way ANOVA with Tukey's multiple comparisons. Source data are provided as a Source Data file.

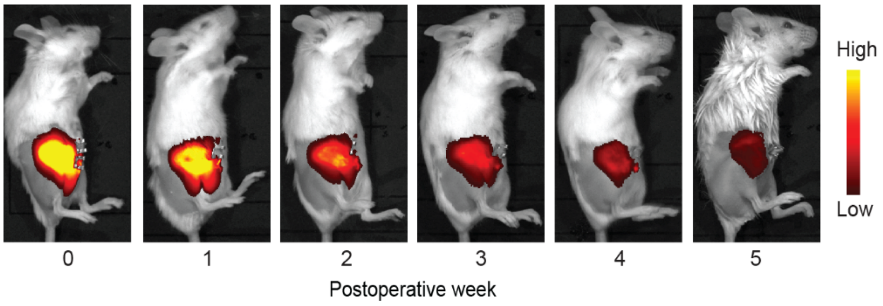

Supplementary Figure 5. In vivo biodistribution of the flex-patch at different time points. Fluorescence IVIS images depicting the sustained retention and disassembly of the implanted patch in vivo. n = 3.

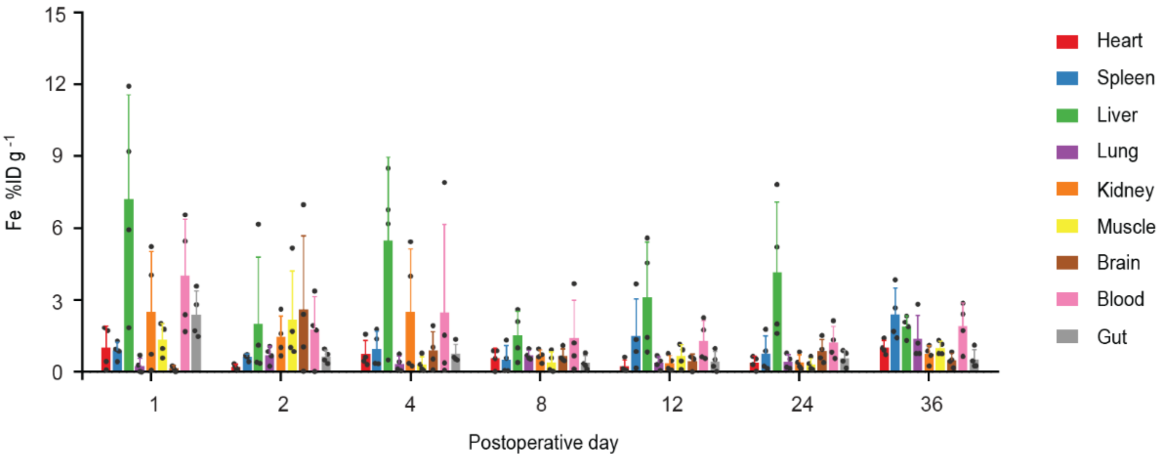

Supplementary Figure 6. In vivo biodistribution of the flex-patch in main tissues of mice at different time points. Concentrations of released items were normalized as the percentage of accumulated dose of Fe per gram of each organ (%ID g^{-1}). n = 4 at each time point. Data are presented as the mean ± SD. Source data are provided as a Source Data file.

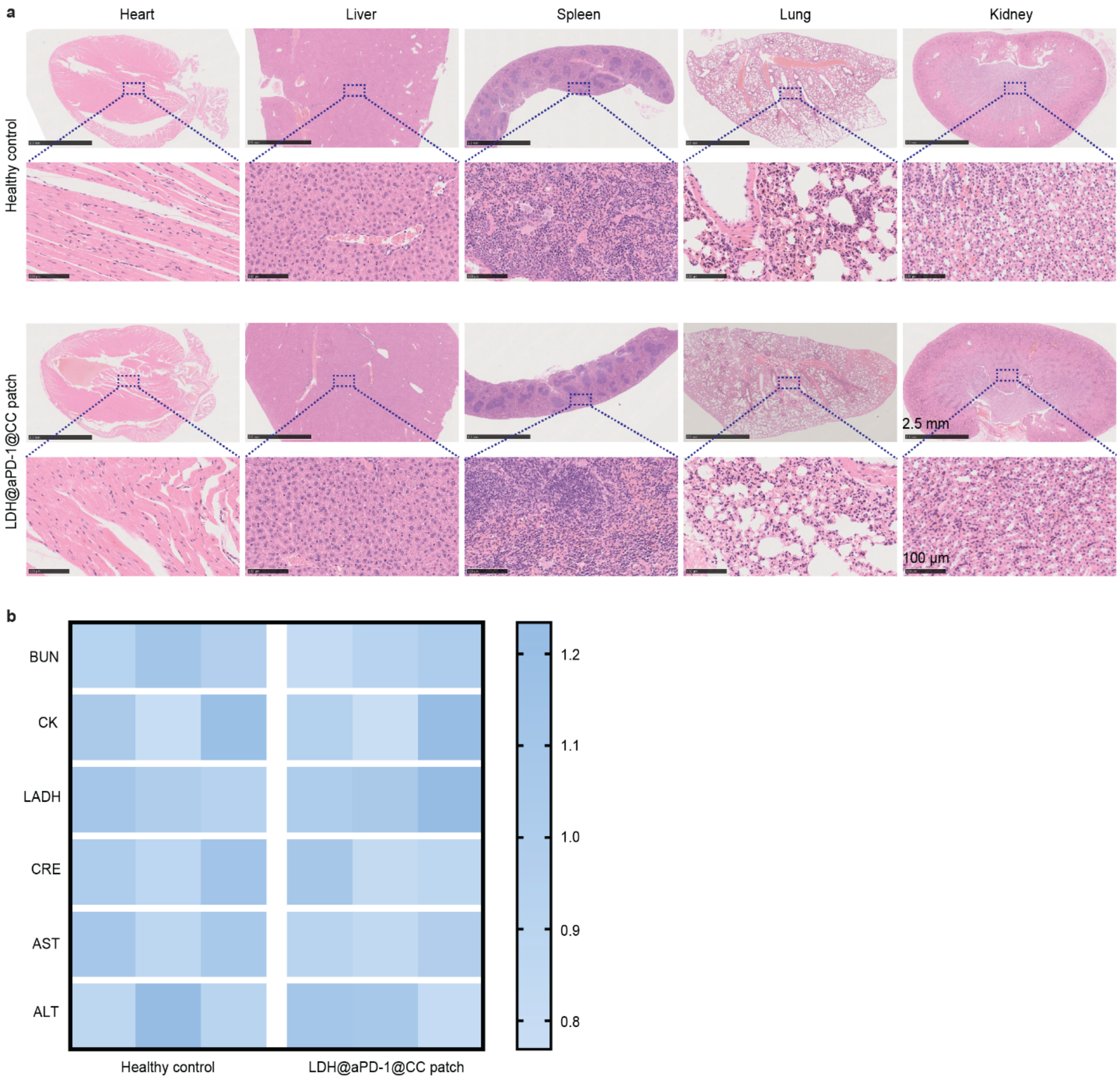

Supplementary Figure 7. In vivo biosafety of the flex-patch. a, Hematoxylin and eosin (H&E) staining of major organs harvested from healthy mice and patch-treated mice on Day 36 postsurgery. Scale bar: 2.5 mm (low-magnification image); scale bar: 100 μm (high-magnification image). **b**, Heatmap exhibiting the relative biochemical levels in murine sera from healthy group and patch-treated group. BUN, blood urea nitrogen; CK, creatine kinase; LADH, lactate dehydrogenase; CRE, creatinine; AST, aspartate transaminase; ALT, alanine aminotransferase. n = 3 per group. Source data are provided as a Source Data file.

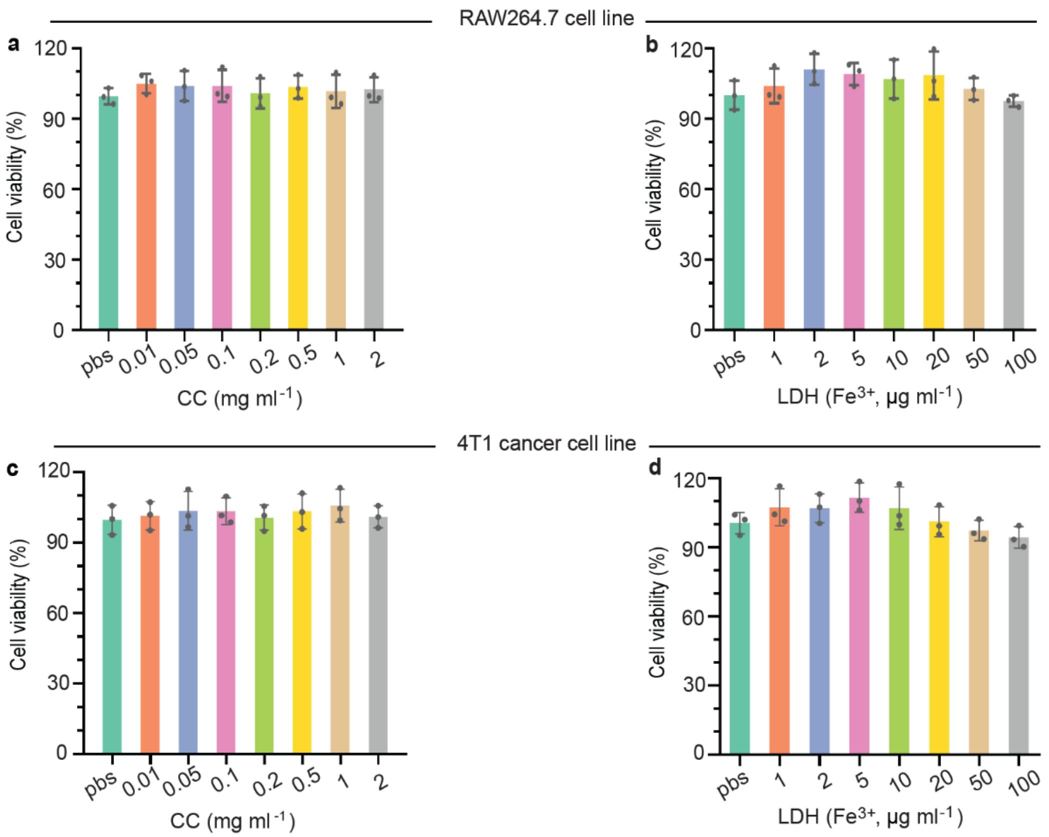

Supplementary Figure 8. Cellular cytotoxicity of the flex-patch. a-d, Cellular cytotoxicity of CC matrix (**a**,**c**) and LDH adjuvant (**b**,**d**), measured by a MTT assay. n = 3 at each concentration point. Data are presented as the mean ± SD. Source data are provided as a Source Data file.

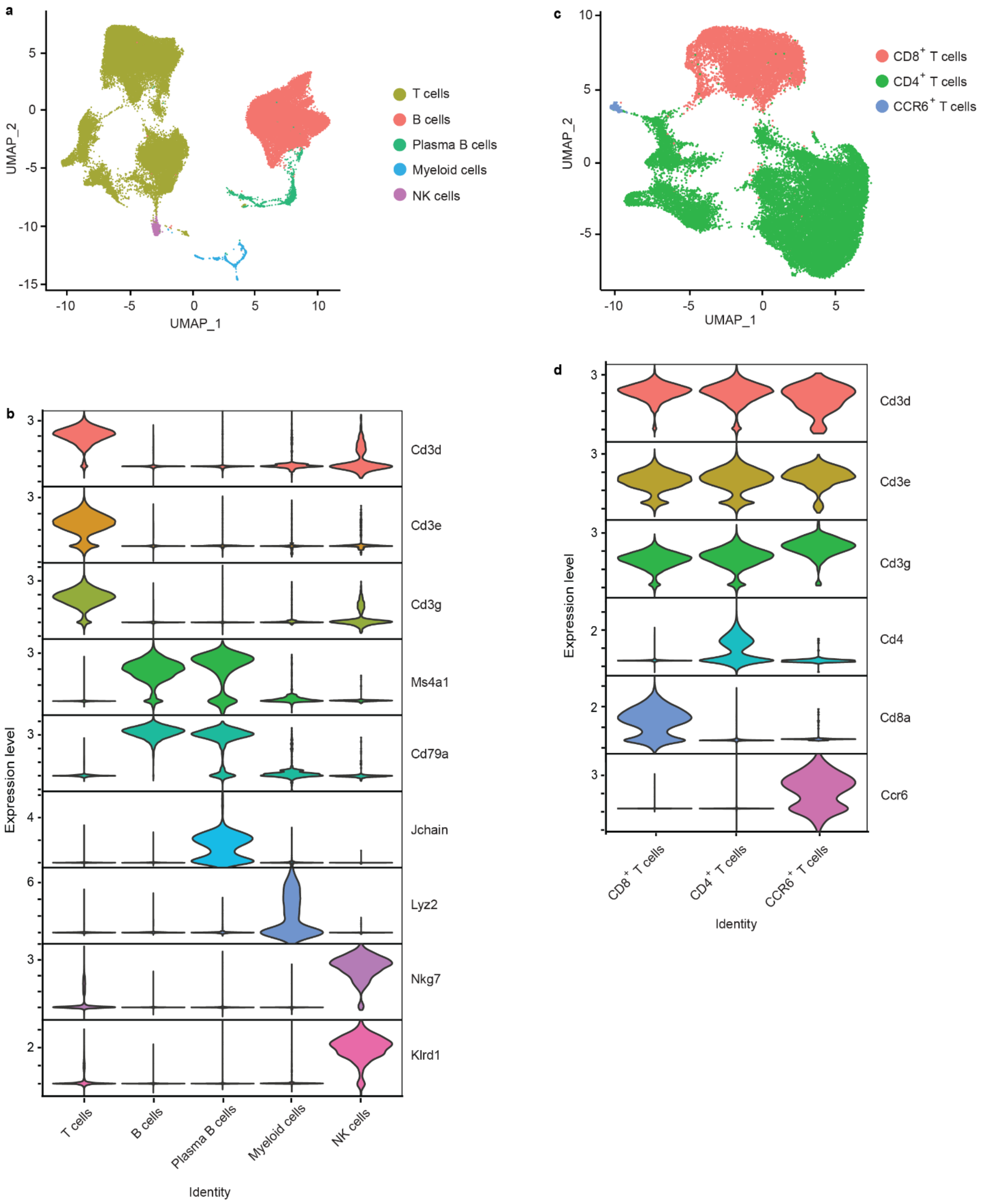

Supplementary Figure 9. Characterization of cell types and T phenotypes in SLN by scRNA seq. a, UMAP plot showing five cell types in SLN. **b**, Violin plots showing cell-type-specific

genes expressed by five cell types in SLN. **c**, UMAP plot showing three color-coded T phenotypes in SLN. **d**, Violin plots showing cell-type-specific genes expressed by three T phenotypes in SLN.

a

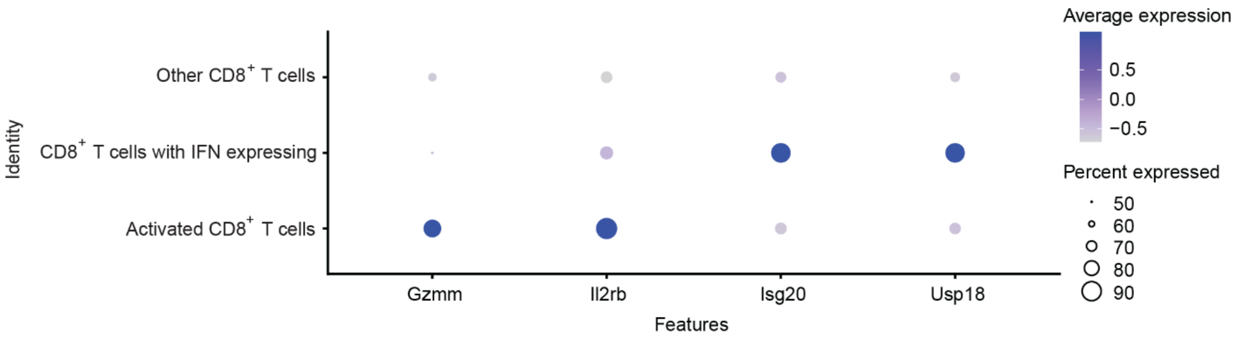

b

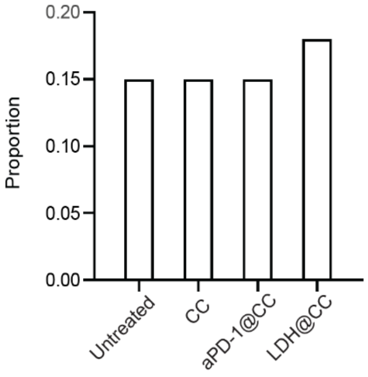

Supplementary Figure 10. CD8$^+$ T subclusters in SLN by scRNA seq. a, Expression heatmap of cell-type-specific genes in two identified CD8$^+$ T subclusters. Columns represent different subclusters and rows represent signature genes. **b**, The proportion of activated CD8$^+$ T cells on total CD8$^+$ T cells per group. Source data are provided as a Source Data file.

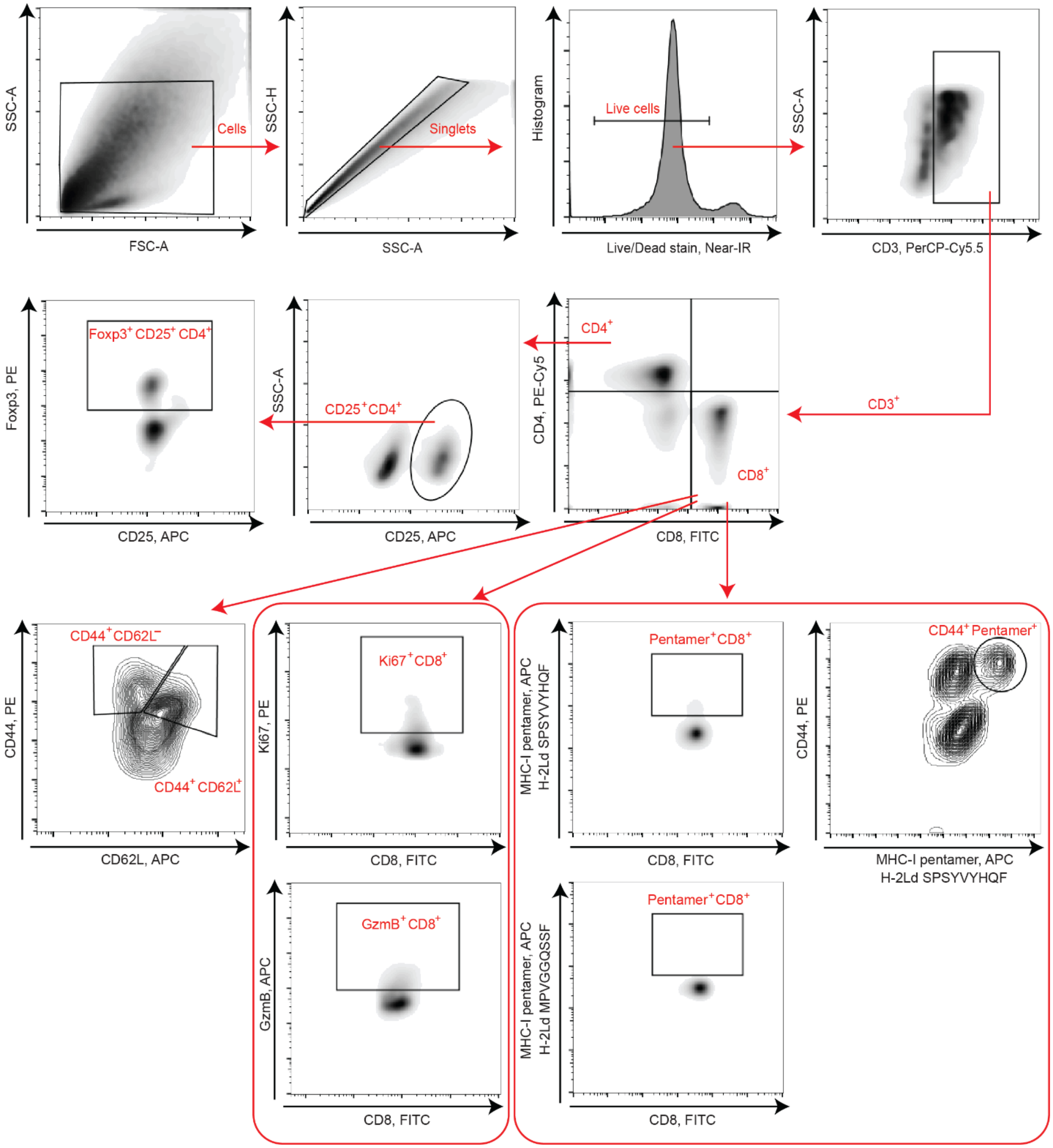

Supplementary Figure 11. Gating strategy in SLN. Representative flow cytometric plots showing the gating strategy to identify $CD8^+$, $CD4^+$ subsets in SLN.

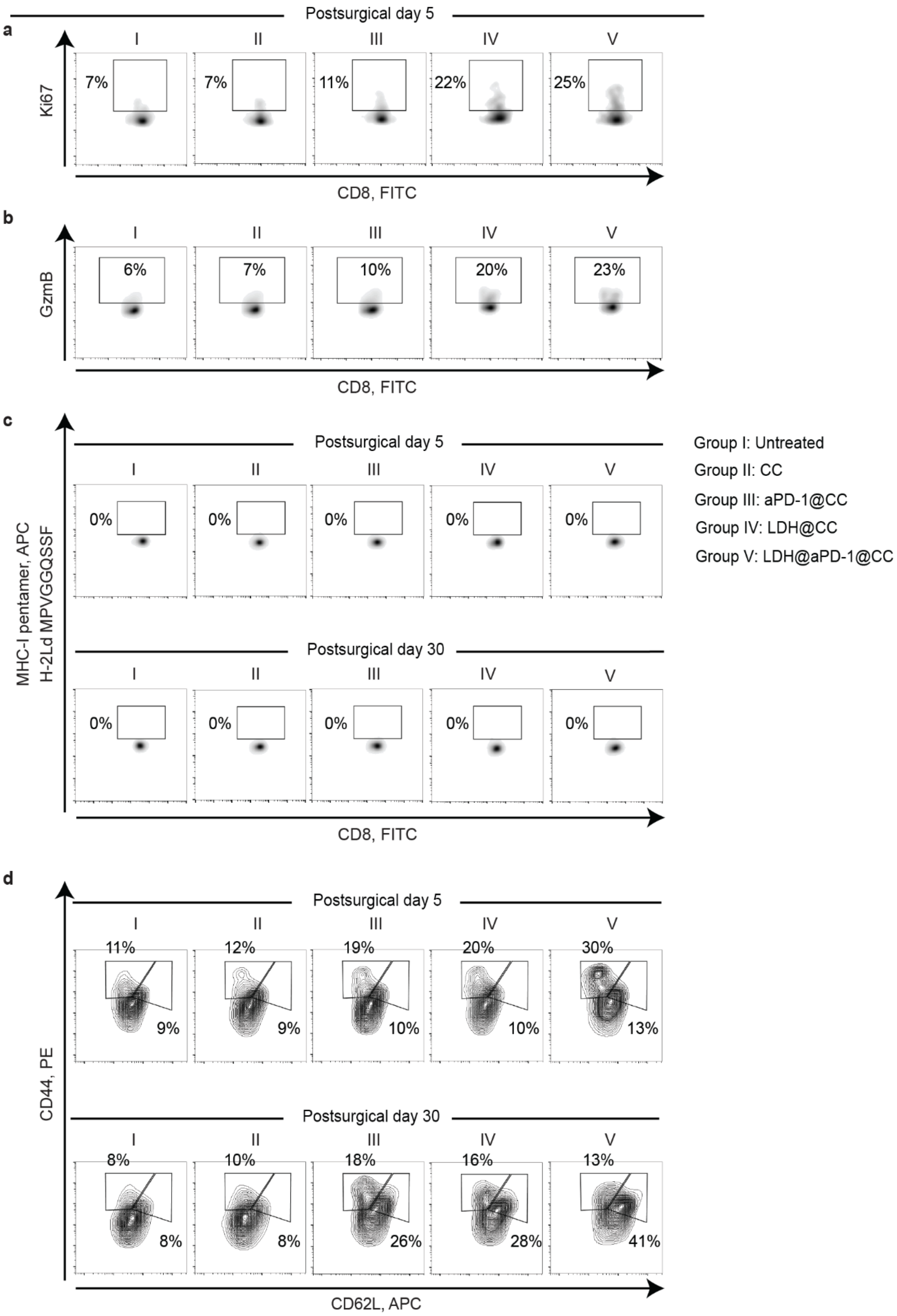
Postsurgical day 5
a
I
II
III
IV
V
Ki67
7%
7%
11%
22%
25%
CD8, FITC
b
GzmB
6%
7%
10%
20%
23%
CD8, FITC
c
Postsurgical day 5
Group I: Untreated
Group II: CC
Group III: aPD-1@CC
Group IV: LDH@CC
Group V: LDH@aPD-1@CC
MHC-I pentamer, APC
H-2Ld MPVGGQSSF
0%
0%
0%
0%
0%
Postsurgical day 30
0%
0%
0%
0%
0%
CD8, FITC
d
Postsurgical day 5
11%
12%
19%
20%
30%
9%
9%
10%
10%
13%
CD44, PE
Postsurgical day 30
8%
10%
18%
16%
13%
8%
8%
26%
28%
41%
CD62L, APC

Supplementary Figure 12. Representative flow cytometric plots of CD8$^+$ subsets in SLN. SLNs in each group were harvested on postsurgical days 5 and 30. **a-d**, Representative flow cytometric plots of Ki67$^+$CD8$^+$ T cells (**a**), GzmB$^+$CD8$^+$ T cells (**b**), H-2Ld MPVGGQSSF-pentamer$^+$CD8$^+$ T cells (**c**), effector memory (T_{em}; CD62L$^-$CD44$^+$) and central memory (T_{cm}; CD62L$^+$CD44$^+$) CD8$^+$ T cells (**d**), gating on CD8$^+$CD3$^+$ cells. n = 5 mice per group.

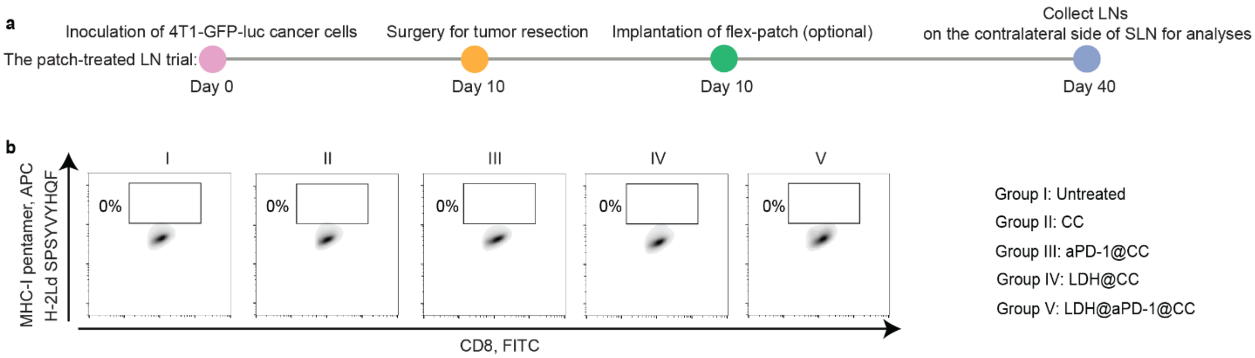

Supplementary Figure 13. Analyses of the LNs on the contralateral side of SLNs. a, Schematic illustrating the LN survey on the 4T1 BC mouse model after surgery, including no patch implantation (untreated, Group I) and patches with different therapeutic agents (CC, Group II; aPD-1@CC, Group III; LDH@CC, Group IV; and LDH@aPD-1@CC, Group V). **b**, Representative flow cytometric plots of H-2Ld SPSYVYHQF-pentamer$^+$CD8$^+$ T cells, gating on CD8$^+$CD3$^+$ cells, on Day 30 postsurgery. n = 5 mice per group.

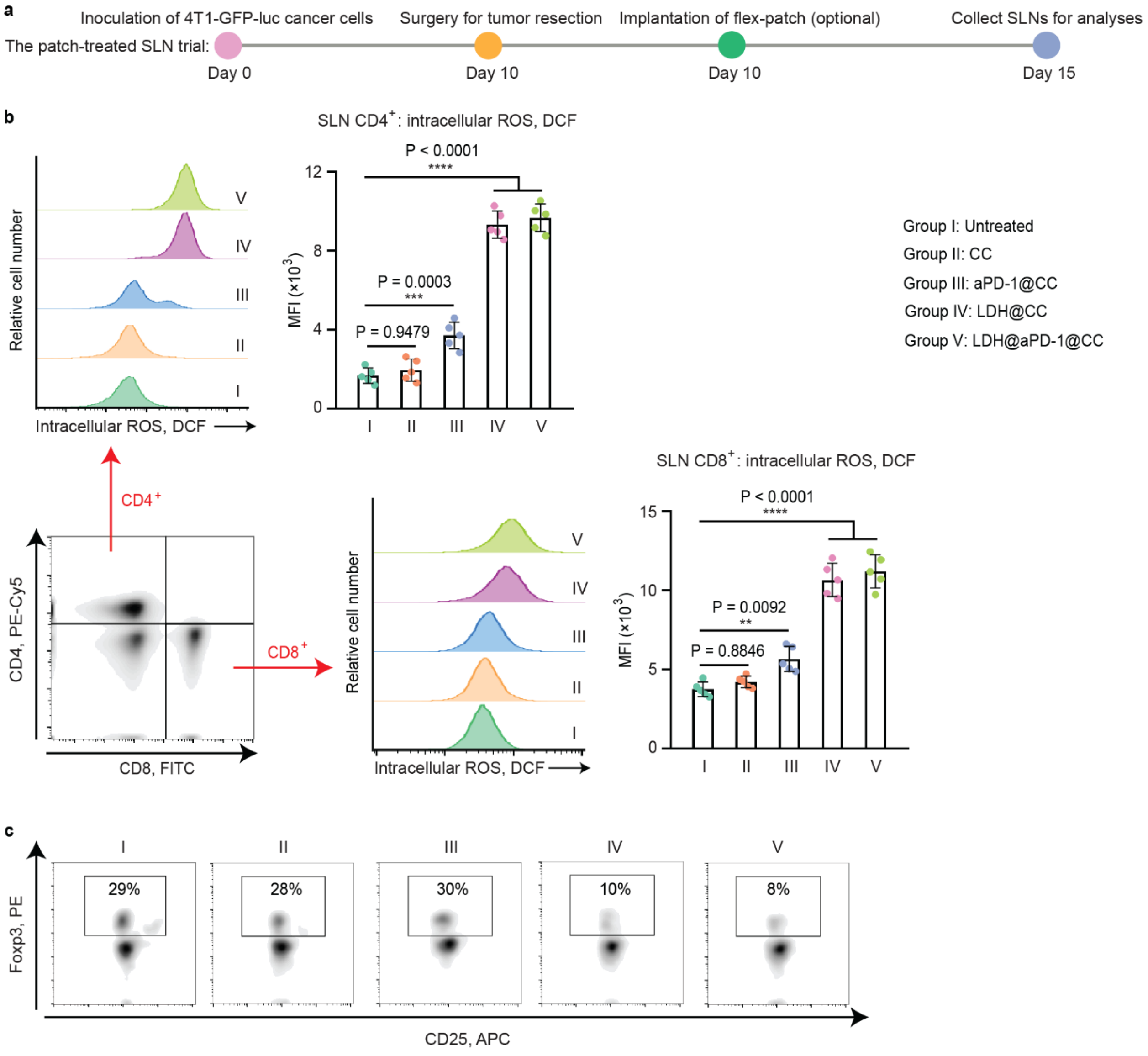

Supplementary Figure 14. ROS-relevant characterizations of patch-treated SLNs. SLNs in each group were harvested on Day 5 postsurgery. **a**, Schematic illustrating the SLN survey on the 4T1 BC mouse model after surgery, including no patch implantation (untreated, Group I) and patches with different therapeutic agents (CC, Group II; aPD-1@CC, Group III; LDH@CC, Group IV; and LDH@aPD-1@CC, Group V). **b**, Representative histograms and quantification of the intracellular ROS levels in $CD8^+$ T cells and $CD4^+$ T cells from SLN. n = 5 mice per group. Data are presented as the mean ± SD, and statistical significance was calculated via one-way ANOVA with Tukey's multiple comparisons. **c**, Representative flow cytometric plots of $Foxp3^+CD25^+CD4^+$ T cells, gating on $CD4^+CD3^+$ cells. n = 5 mice per group. Source data are provided as a Source Data file.

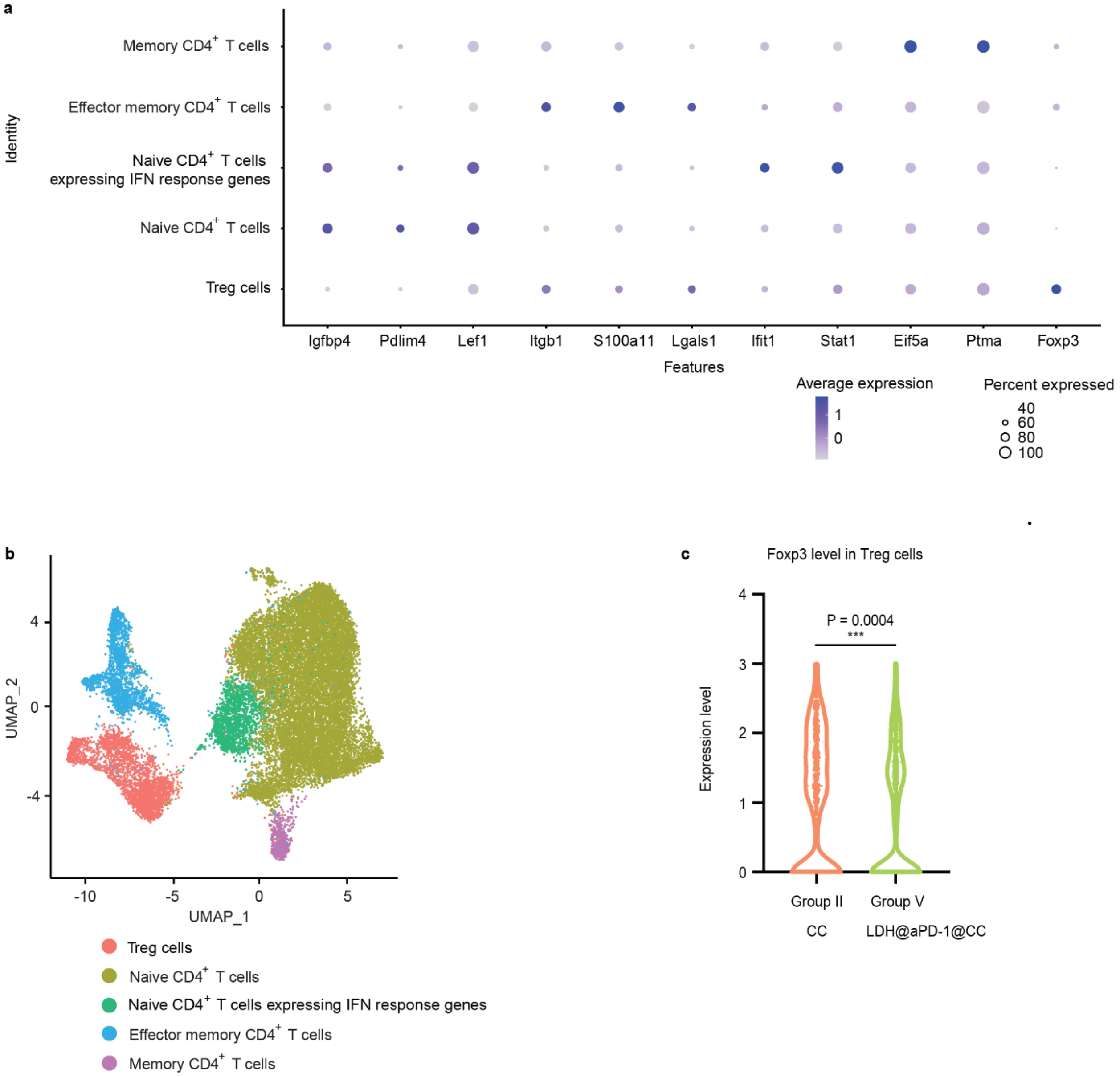

Supplementary Figure 15. Analyses of Treg cells by scRNA seq. a,**b**, Expression heatmap of cell-type-specific genes in five $CD4^+$ T subclusters (**a**) with their UMAP plot (**b**). Columns represent different subclusters and rows represent signature genes. **c**, Foxp3 expression level in Treg cells. Statistical significance was calculated via unpaired two-tailed t-test. Source data are provided as a Source Data file.

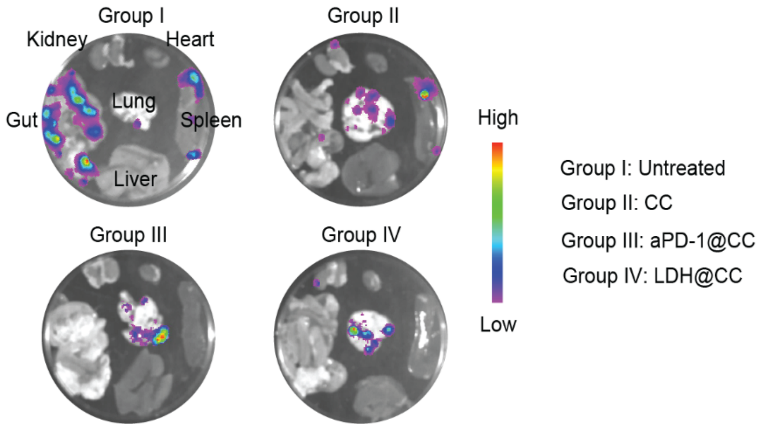

Supplementary Figure 16. Postsurgical 4T1-BC treatment of flex-patch. Representative bioluminescence images of visceral organs of tumor mice in groups I-IV.

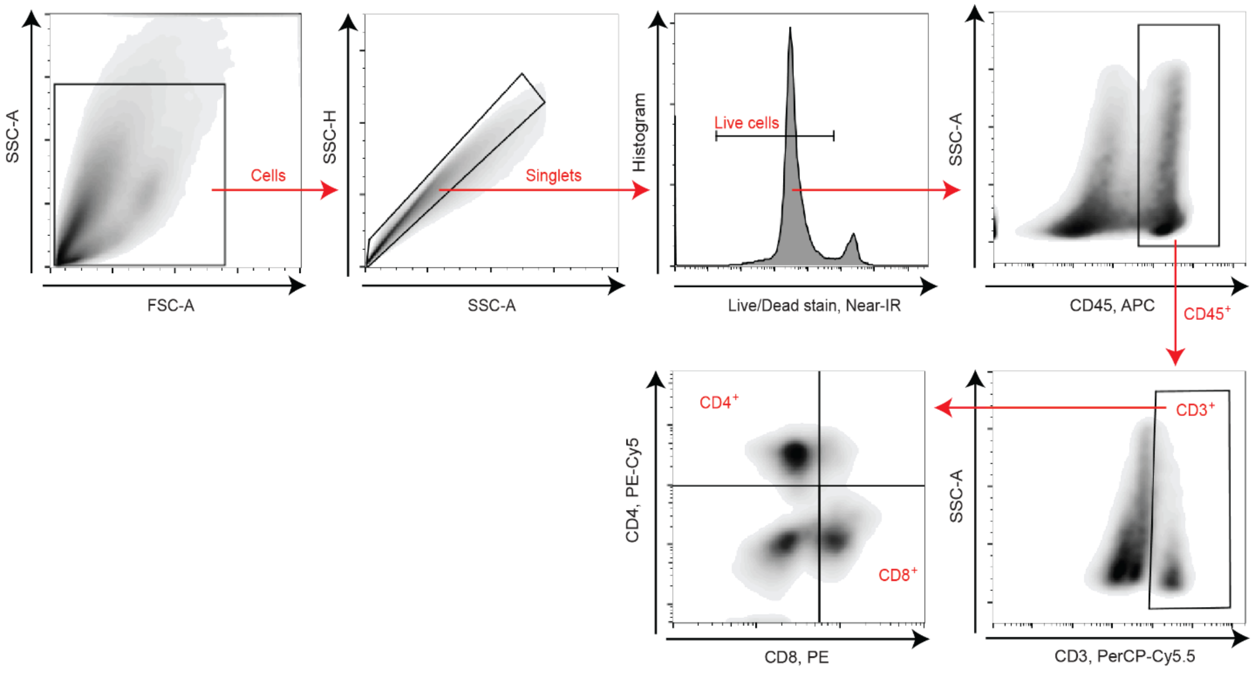

Supplementary Figure 17. Gating strategy in postsurgical 4T1 tumor. Representative flow cytometric plots showing the gating strategy used to identify $CD8^{+}CD3^{+}CD45^{+}$ in postsurgical 4T1 tumor.

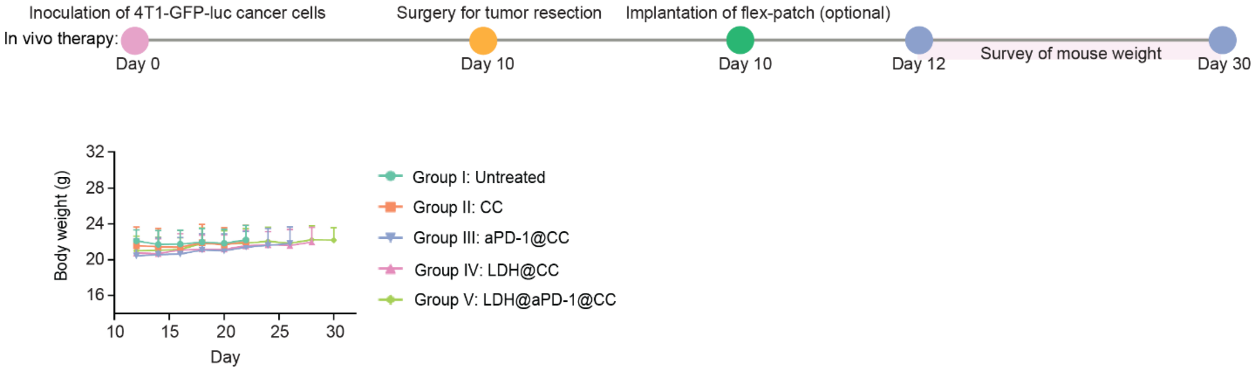

Supplementary Figure 18. Therapeutic influence of flex-patch on the weight of 4T1 BC mice. Weight changes of 4T1 BC mice after the surgery and optional patch implantation detailed in the schematic process, including no patch (untreated, Group I) and patches with different therapeutic agents (CC, Group II; aPD-1@CC, Group III; LDH@CC, Group IV; and LDH@aPD-1@CC, Group V). n = 12 mice per group. Weighing stopped when the first mouse in the group was euthanized. Data are presented as the mean ± SD. Source data are provided as a Source Data file.

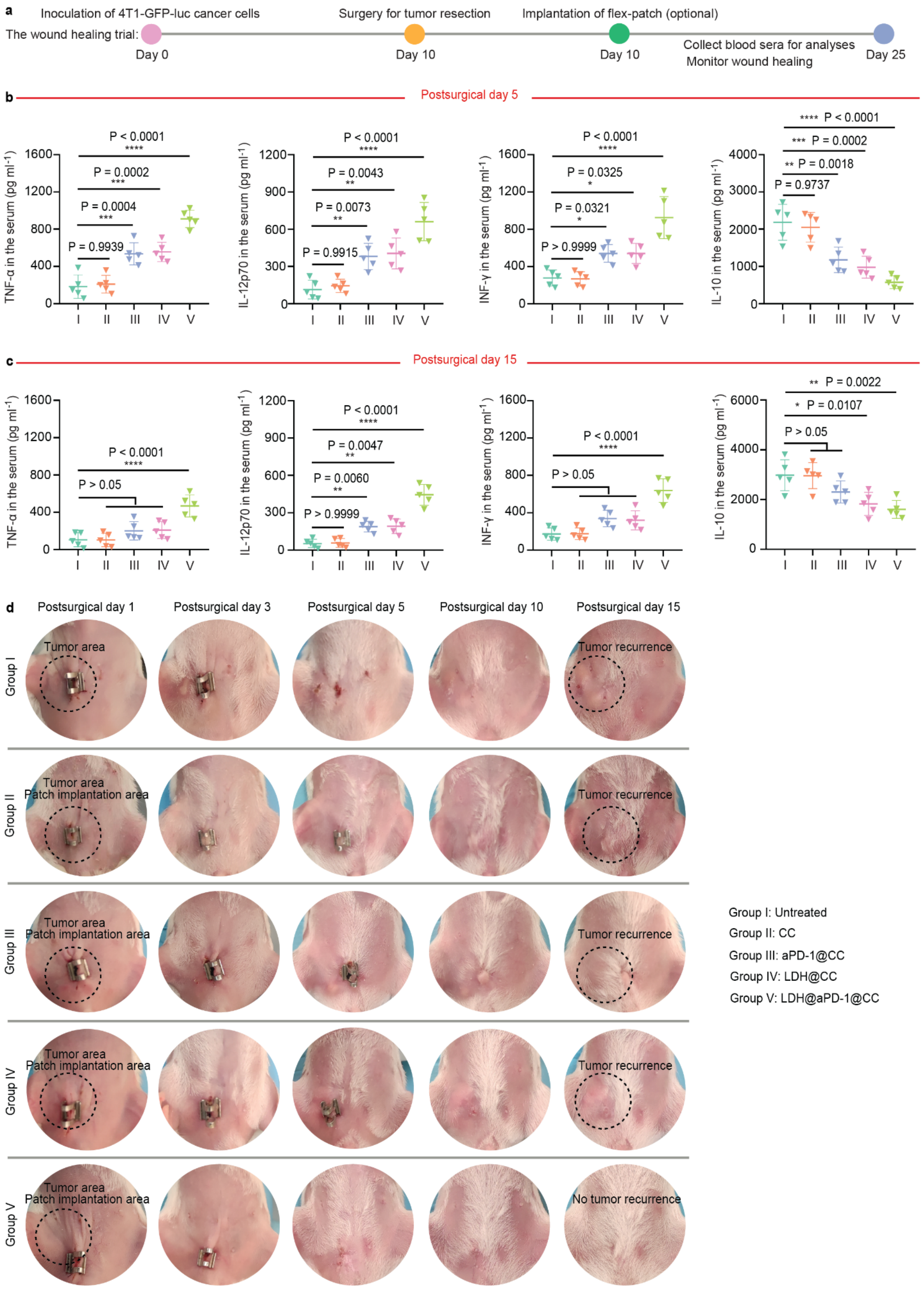
a
Inoculation of 4T1-GFP-luc cancer cells
Surgery for tumor resection
Implantation of flex-patch (optional)
The wound healing trial:
Day 0
Day 10
Day 10
Collect blood sera for analyses
Monitor wound healing
Day 25
b
Postsurgical day 5
TNF-α in the serum (pg ml-1)
IL-12p70 in the serum (pg ml-1)
INF-γ in the serum (pg ml-1)
IL-10 in the serum (pg ml-1)
c
Postsurgical day 15
d
Postsurgical day 1
Postsurgical day 3
Postsurgical day 5
Postsurgical day 10
Postsurgical day 15
Group I
Group II
Group III
Group IV
Group V
Tumor area
Patch implantation area
Tumor recurrence
No tumor recurrence
Group I: Untreated
Group II: CC
Group III: aPD-1@CC
Group IV: LDH@CC
Group V: LDH@aPD-1@CC

Supplementary Figure 19. Analyses of hemal cytokines and postsurgical wound. a, Schematic illustrating the route of blood sera assay and wound-healing monitoring in the 4T1 BC mouse model after surgery, including no patch implantation (untreated, Group I) and patches with different therapeutic agents (CC, Group II; aPD-1@CC, Group III; LDH@CC, Group IV; and LDH@aPD-1@CC, Group V). n = 5 mice per group. **b**,**c**, Hematic TNF-α, IL-12, IFN-γ, and IL-10 levels on Day 5 (**b**) and Day 15 (**c**) postsurgery. 100 μL of blood per mouse was collected on each time point through tail vein. n = 5 mice per group; data are presented as the mean ± SD; statistical significance was calculated via one-way ANOVA with Tukey's multiple comparisons. **d**, Photographs of postsurgical wound per mouse during the treating process in (**a**). Source data are provided as a Source Data file.

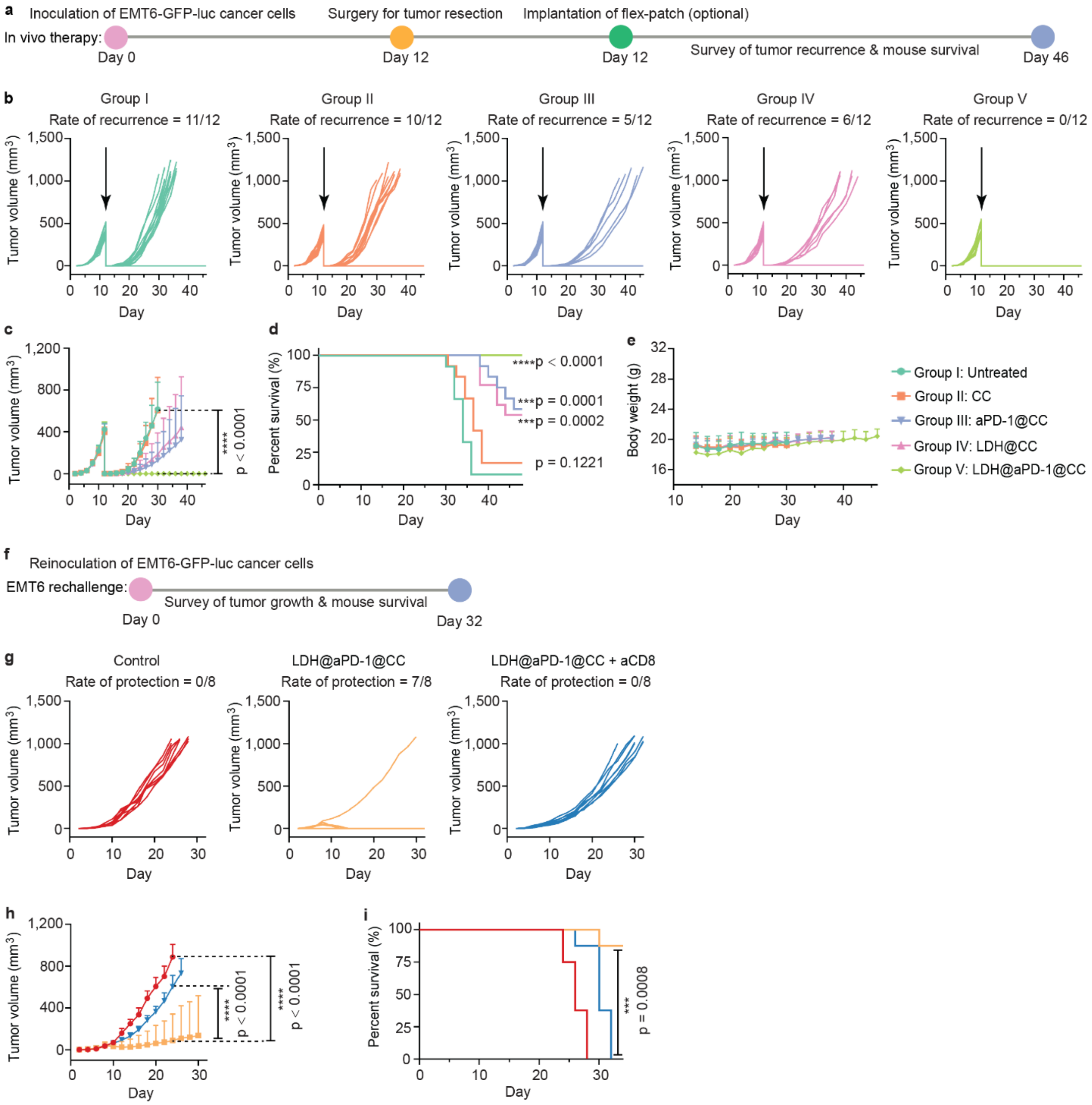

Supplementary Figure 20. Postsurgical EMT6-BC treatment of flex-patch. a, Schematic illustrating therapeutic procedure on the EMT6 BC mouse model after surgery, including no patch implantation (untreated, Group I) and patches with different therapeutic agents (CC, Group II; aPD-1@CC, Group III; LDH@CC, Group IV; and LDH@aPD-1@CC, Group V). n = 12 mice per group. **b**,**c**, Individual (**b**) and average (**c**) tumor growth kinetics per group. **d**,**e**, Mouse survival (**d**) and weight changes (**e**) per group. **f**, Schematic illustrating the route of the EMT6 tumor rechallenge assay. Apart from the control group, all tumor-free mice were harvested from Group

V (LDH@aPD-1@CC). n = 8 mice per group. **g**,**h**, Individual (**g**) and average (**h**) tumor growth kinetics per group. **i**, Mouse survival in different rechallenge groups. For (**c**,**e**,**h**), the curve ended when the first mouse in the corresponding group died. Data are presented as the mean ± SD. Statistical significance was calculated via one-way ANOVA with Tukey's multiple comparisons. For (**d**,**i)**, statistical significance was calculated via the log-rank (Mantel–Cox) test by comparison with the untreated Group I (or the control group). Source data are provided as a Source Data file.

Supplementary Table 1. Table of chemical reagents and functional kits

**Reagent**	**Cat #**	**Supplier**
Magnesium nitrate hexahydrate	13446-18-9	Sigma-Aldrich
Magnesium chloride hexahydrate	7791-18-6	Sigma-Aldrich
Iron(III) nitrate nonahydrate	7782-61-8	Sigma-Aldrich
Iron(III) chloride hexahydrate	10025-77-1	Sigma-Aldrich
Sodium hydroxide	1310-73-2	Sigma-Aldrich
Carboxylated chitosan	9012-76-4	Aladdin
Avertin	75-80-9	Sigma-Aldrich
Sodium Pyruvate	P4562-25g	Sigma-Aldrich
β-Mercaptoethanol	M3148	Sigma-Aldrich
L-Glutathione reduced	G6013-10G	Sigma-Aldrich
Phorbol 12-myristate 13-acetate (PMA)	P849986-1mg	Macklin
Ionomycin	I838446-1mg	Macklin
Poly-D-lysine	E607014	Sangon Biotech
2’,7’-Dichlorofluorescein diacetate (DCFHDA)	35845	Sigma-Aldrich
Mouse IL-2 ELISA kit	LEM020-2	Laizee
Mouse IgG ELISA kit	EMC116(H)	Invitrogen
Mouse IL-10 ELISA kit	EMC005.95.2	NeoBioscience
Mouse TNF-α ELISA kit	EMC102a.96.2	NeoBioscience
Mouse IL-6 ELISA kit	EMC004.96.2	NeoBioscience
Mouse IL-12p70 ELISA kit	EMC006.96.2	NeoBioscience
Mouse IFN-γ ELISA kit	EMC101g.96.2	NeoBioscience
EasySep™ Mouse $CD8^+$ T cell isolation kit	19853	Stemcell
CytoTox 96® non-radioactive cytotoxicity assay kit	G1780	Promega

Nuclear and cytoplasmic protein extraction kit	P0028	Beyotime
QuantiTect reverse transcription kit	205311	Qiagen
Pierce BCA protein assay kit	23225	Thermo Fisher Scientific
Glutathione assay kit	703002	Cayman Chemical
LIVE/DEAD™ fixable near-IR dead cell stain kit	L10119	Invitrogen
BUN assay kit	C013-2-1	NanJing JianCheng Bioengineering Institute
CRE assay kit	C011-2-1	NanJing JianCheng Bioengineering Institute
CK assay kit	A032-1-1	NanJing JianCheng Bioengineering Institute
LADH assay kit	A020-1	NanJing JianCheng Bioengineering Institute
AST assay kit	C010-2-1	NanJing JianCheng Bioengineering Institute
ALT assay kit	C009-2-1	NanJing JianCheng Bioengineering Institute

Supplementary Table 2. Table of antibodies

Target	Cat #	Clone	Supplier	Fluor	Dilution ratio
PD-1	P372	RMP1-14	Leinco	none	No dilution (used for the flex-patch design)
PD-1	11-9985-82	J43	eBioscience	yes	1:50
PD-1	17-9981-82	RMP1-30	eBioscience	yes	1:20
PD-1	12-9985-82	J43	eBioscience	yes	1:40
PD-1	ab214421	EPR20665	abcam	none	1:200
CD16/32	156604	S17011E	BioLegend	none	1:200
CD8	F398-84A-G	KT15	Proimmune	yes	1:60
CD8	45-0081-82	53-6.7	eBioscience	yes	1:80
CD8	12-0081-83	53-6.7	eBioscience	yes	1:80
CD8	85336	D8A8Y	Cell signaling technology	none	1:400
CD3	45-0031-82	145-2C11	eBioscience	yes	1:20
CD3	11-0031-85	145-2C11	eBioscience	yes	1:100
CD3	100340	145-2C11	Biolegend	none	1:492
CD3	78588	E4T1B	Cell signaling technology	none	1:400
Ki67	652404	16A8	BioLegend	yes	1:50
Ki67	ab16667	SP6	abcam	none	1:300
Granzyme B	372204	QA16A02	BioLegend	yes	1:20
CD44	12-0441-82	IM7	eBioscience	yes	1:150
CD62L	17-0621-82	MEL-14	eBioscience	yes	1:300
CD62L	25-0621-82	MEL-14	eBioscience	yes	1:80
CD4	17-0041-83	GK1.5	eBioscience	yes	1:150
CD4	15-0041-83	GK1.5	eBioscience	yes	1:300

Foxp3	12-5773-82	FJK-16s	eBioscience	yes	1:20
CD45	17-0451-82	30-F11	eBioscience	yes	1:150
CD45	45-0451-82	30-F11	eBioscience	yes	1:150
NFAT1	MA1-025	25A10.D6.D2	Invitrogen	none	1:1000
PCNA	ARG62605	PC10	Arigo biolaboratories	none	1:1000
Cleaved caspase-3 (CC3)	9661	Asp175	Cell signaling technology	none	1:500
GFP	sc-9996	B-2	Santa cruz biotechnology	none	1:200
β-actin	3700	8H10D10	Cell signaling technology	none	1:2000
CD28	553295	37.51	BD Pharmingen	none	1:200
CD25	20-0251-U100	PC61.5	Tonbo Bioscience	yes	1:600
CD69	12-0691-82	H1.2F3	eBioscience	yes	1:350
CD107a	121614	1D4B	BioLegend	yes	1:20
Phospho-FAK (Tyr397)	700255	31H5L17	Invitrogen	none	1:200
Phospho-ERK1/2 (Thr202/Tyr204)	369504	6B8B69	BioLegend	yes	1:20

Supplementary Table 3. Table of cell lines and products for cell culture

**Product name**	**Cat #**	**Supplier**
4T1 cell line	CRL-2539	the American Type Culture Collection
4T1-GFP-luc cell line		generated by Dr. Kai Miao at University of Macau
EMT6 cell line	CRL-2755	the American Type Culture Collection
EMT6-GFP-luc cell line		generated by Dr. Kai Miao at University of Macau
RAW264.7 cell line	TIB-71	the American Type Culture Collection
B16F10 cell line	CRL-6475	the American Type Culture Collection
Roswell Park Memorial Institute 1640 Medium	11875-093	Gibco
Dulbecco's Modified Eagle's Medium	11965-092	Gibco
DMEM/F-12 Medium	11330032	Gibco
Fetal bovine serum	26140079	Gibco
Penicillin-streptomycin	15140122	Gibco

Supplementary Table 4. Table of bioactive products and consumables

**Product name**	**Cat #**	**Supplier**
Pro5 MHC-I pentamer (H-2Ld SPSYVYHQF)	F398-84A-G	Proimmune
Pro5 MHC-I pentamer (H-2Ld MPVGGQSSF)	F158-84A-G	Proimmune
SPSYVYHQF peptide		custom-made in Suzhou Bestbiochem Pharma-tech Co., Ltd
FastStart universal SYBR green master	4913850001	Roche Diagnostics
VivoGlo™ Luciferin	P1043	Promega
Collagenase type III	LS0004182	Worthington
Hyaluronidase	H3506	Sigma-Aldrich
Insulin	I1882	Sigma-Aldrich
Epidermal growth factor protein	PHG0311L	Gibco
Hydrocortisone	H0888	Sigma-Aldrich
Dispase II	04942078001	Roche
DNase I	58C10349	Worthington
Red blood cell lysis buffer	00433357	Invitrogen
Foxp3/transcription factor staining buffer set	00-5523-00	eBioscience
PF-562271 (FAK inhibitor)	S2890	Selleckchem
11R-VIVIT TFA (NFAT inhibitor)	P1210	Selleckchem
Slide-A-Lyzer MINI dialysis device (3.5K MWCO)	88400	Thermo Fisher Scientific
Snakeskin dialysis membrane	68035	Thermo Fisher Scientific
12-well Transwell plate	3462	Costar
70-µm cell strainer	52350	Falcon

References

1. Choy, J. H., Kwak, S. Y., Jeong, Y. J. & Park, J. S. Inorganic layered double hydroxides as nonviral vectors. *Angew. Chem. Int. Ed.* **39**, 4042-4045 (2000).
2. King, B. F. et al. Antagonism of ATP responses at P2X receptor subtypes by the pH indicator dye, phenol red. *Brit. J. Pharmacol.* **145**, 313-322 (2005).
