# Supplementary material for: Fueling sentinel node via reshaping cytotoxic T lymphocytes with a flex-patch for post-operative immuno-adjuvant therapy
Source: Nat Commun. 2023 May 2;14:2518. doi: 10.1038/s41467-023-38245-7 (PMC10154421; doi:10.1038/s41467-023-38245-7)
Supplement: Supplementary file 3 — Reporting Summary [file 41467_2023_38245_MOESM3_ESM.pdf]

nature portfolio

Corresponding author(s): Dr. Yunlu Dai

Last updated by author(s): Mar 30, 2023

Reporting Summary

Nature Portfolio wishes to improve the reproducibility of the work that we publish. This form provides structure for consistency and transparency in reporting. For further information on Nature Portfolio policies, see our Editorial Policies and the Editorial Policy Checklist.

Statistics

For all statistical analyses, confirm that the following items are present in the figure legend, table legend, main text, or Methods section.

n/a	Confirmed	
☐	☒	The exact sample size (*n*) for each experimental group/condition, given as a discrete number and unit of measurement
☐	☒	A statement on whether measurements were taken from distinct samples or whether the same sample was measured repeatedly
☐	☒	The statistical test(s) used AND whether they are one- or two-sided<br>*Only common tests should be described solely by name; describe more complex techniques in the Methods section.*
☒	☐	A description of all covariates tested
☒	☐	A description of any assumptions or corrections, such as tests of normality and adjustment for multiple comparisons
☐	☒	A full description of the statistical parameters including central tendency (e.g. means) or other basic estimates (e.g. regression coefficient) AND variation (e.g. standard deviation) or associated estimates of uncertainty (e.g. confidence intervals)
☐	☒	For null hypothesis testing, the test statistic (e.g. *F*, *t*, *r*) with confidence intervals, effect sizes, degrees of freedom and *P* value noted<br>*Give P values as exact values whenever suitable.*
☒	☐	For Bayesian analysis, information on the choice of priors and Markov chain Monte Carlo settings
☒	☐	For hierarchical and complex designs, identification of the appropriate level for tests and full reporting of outcomes
☒	☐	Estimates of effect sizes (e.g. Cohen's *d*, Pearson's *r*), indicating how they were calculated

Our web collection on statistics for biologists contains articles on many of the points above.

Software and code

Policy information about availability of computer code

Data collection	The morphology, composition, and disassembling of the flex-patch were characterized by scanning electron microscopy (Hitachi S-3400N, Type I) and energy dispersive X-ray spectroscopy (Horiba EX-250), transmission electron microscopy (Jeol JEM-ARM300F), Zetasizer 8.01 (Malvern Panalytical), X-ray powder diffraction, Fourier transform infrared (IRAffinity-1S, SHIMADZU) spectroscopy, Leica DFC450 C, and Thermo iCAP Qs inductively coupled plasma mass spectrometry. The implanting process of the flex-patch was imaged by Leica DFC450 C microscopy. The in vivo biodistribution of the flex-patch was imaged and quantified using an IVIS Spectrum Imaging system (Perkin Elmer). Data on immunohistochemistry (IHC) and hematoxylin and eosin (H&E) staining of mouse tumors and lymph nodes were obtained using NanoZoomer S60 Digital slide scanner (C13210-01).Tumor luminescence from mouse or mouse organs was imaged by an in vivo Xtreme 4MP system (Bruker MI) with the "Luminescence" modality and "Reflectance" background image. Flow cytometry data were collected using the CytoFLEX S flow cytometer (Beckman). To collect data on glycolytic activity, Seahorse XF96 extracellular flux analyzer (Agilent Technologies) was applied. The scRNA seq data were acquired using CellRanger tookit (v4.0.0, 10x Genomics). Western blot images were collected using Bio-Rad Image lab 6.1.
Data analysis	All statistical analyses were performed on Graphpad Prism (v8.0). IHC and H&E slices were analyzed by SlideViewer 2.5. All flow cytometry data were analyzed on FlowJo software (v10.0.7). The scRNA seq data were processed using SingleR (v1.4.0). Also, FindMarkers and database searching confirmed the marker genes per cluster. R package DESeq2(v.1.30.0) was applied to analyze different expression genes (DEGs). Gene set enrichment analysis (GSEA) was applied to identify metabolic pathways enriched in genes with highest variability. GSEA analysis was done by the software javaGSEA available at https://www.gsea-msigdb.org/gsea/index.jsp.

For manuscripts utilizing custom algorithms or software that are central to the research but not yet described in published literature, software must be made available to editors and reviewers. We strongly encourage code deposition in a community repository (e.g. GitHub). See the Nature Portfolio guidelines for submitting code & software for further information.

Data

Policy information about availability of data

All manuscripts must include a data availability statement. This statement should provide the following information, where applicable:

- Accession codes, unique identifiers, or web links for publicly available datasets
- A description of any restrictions on data availability
- For clinical datasets or third party data, please ensure that the statement adheres to our policy

The single-cell RNA sequencing data generated in this study have been deposited in the Sequence Read Archive database with the accession code of PRJNA853539. Source data are provided with this paper.

Human research participants

Policy information about studies involving human research participants and Sex and Gender in Research.

Reporting on sex and gender	This study was not sex/gender-specific. Although breast cancer is generally diagnosed among females, males can develop it.
Population characteristics	This study was not sex/gender-specific.
Recruitment	This study was not sex/gender-specific.
Ethics oversight	This study was not sex/gender-specific.

Note that full information on the approval of the study protocol must also be provided in the manuscript.

Field-specific reporting

Please select the one below that is the best fit for your research. If you are not sure, read the appropriate sections before making your selection.

[x] Life sciences [ ] Behavioural & social sciences [ ] Ecological, evolutionary & environmental sciences

For a reference copy of the document with all sections, see nature.com/documents/nr-reporting-summary-flat.pdf

Life sciences study design

All studies must disclose on these points even when the disclosure is negative.

Sample size	Sample size was approximately based on previous studies (PMID: 33903744, 33526838) in the field, pilot experiments, and reviewer's suggestion. A reasonable sample size was estimated to perform valid statistical analysis and to ensure the reproducibility of the results. Exact sample size (n number) information was provided in the figure legends.
Data exclusions	Cells of poor quality in scRNA seq samples were excluded if cells with < 200 or > 6,000 detected genes. Cells with high-level mitochondrial gene expression were also filtered out through estimating a median-absolute deviation (MAD) variance with median centered. The potential doublets were identified and removed.
Replication	$n \geq 3$ was used for all experiments to ensure that the experimental results were reliable. Biological and technical replicates were considered. Multiple assays including scRNA seq analysis, glycolytic assay, and flow cytometry analysis were performed to cross-validate key findings. Two independent breast tumor models were applied to validate key data.
Randomization	For in vivo tests, same-age mice after tumor inoculation were randomly allocated to experimental groups before the surgical removal of the tumor. All samples collected from each individual mouse per group were analyzed together for each experiment using the same processing and analysis method.
Blinding	Investigators were blinded to group collection during data acquisition and analysis for in vivo experiments. Blinding for in vitro experiments was not required as same parameter and analysis method was adopted to all the experimental conditions during data acquisition and analysis.

Reporting for specific materials, systems and methods

We require information from authors about some types of materials, experimental systems and methods used in many studies. Here, indicate whether each material, system or method listed is relevant to your study. If you are not sure if a list item applies to your research, read the appropriate section before selecting a response.

Materials & experimental systems

n/a	Involved in the study	
[ ]	[x]	Antibodies
[ ]	[x]	Eukaryotic cell lines
[x]	[ ]	Palaeontology and archaeology
[ ]	[x]	Animals and other organisms
[x]	[ ]	Clinical data
[x]	[ ]	Dual use research of concern

Methods

n/a	Involved in the study	
[x]	[ ]	ChIP-seq
[ ]	[x]	Flow cytometry
[x]	[ ]	MRI-based neuroimaging

Antibodies

Antibodies used

1) Anti-PD-1, P372, clone RMP1-14, Leinco, no dilution (used for the flex-patch design);
2) Anti-PD-1, 11-9985-82, clone J43, eBioscience, dilution ratio 1:50;
3) Anti-PD-1, 17-9981-82, clone RMP1-30, eBioscience, dilution ratio 1:20;
4) Anti-PD-1, 12-9985-82, clone J43, eBioscience, dilution ratio 1:40;
5) Anti-PD-1, ab214421, clone EPR20665, abcam, dilution ratio 1:200;
6) Anti-CD16/32, 156604, clone S17011E, BioLegend, dilution ratio 1:200;
7) Anti-CD8, F398-84A-G, clone KT15, Proimmune, dilution ratio 1:60;
8) Anti-CD8, 45-0081-82, clone 53-6.7, eBioscience, dilution ratio 1:80;
9) Anti-CD8, 12-0081-83, clone 53-6.7, eBioscience, dilution ratio 1:80;
10) Anti-CD8, 85336, clone D8A8Y, Cell signaling technology, dilution ratio 1:400;
11) Anti-CD3, 45-0031-82, clone 145-2C11, eBioscience, dilution ratio 1:20;
12) Anti-CD3, 11-0031-85, clone 145-2C11, eBioscience, dilution ratio 1:100;
13) Anti-CD3, 100340, clone 145-2C11, Biolegend, dilution ratio 1:492;
14) Anti-CD3, 78588, clone E4T1B, Cell signaling technology, dilution ratio 1:400;
15) Anti-Ki67, 652404, clone 16A8, BioLegend, dilution ratio 1:50;
16) Anti-Ki67, ab16667, clone SP6, abcam, dilution ratio 1:300;
17) Anti-Granzyme B, 372204, clone QA16A02, BioLegend, dilution ratio 1:20;
18) Anti-CD44, 12-0441-82, clone IM7, eBioscience, dilution ratio 1:150;
19) Anti-CD62L, 17-0621-82, clone MEL-14, eBioscience, dilution ratio 1:300;
20) Anti-CD62L, 25-0621-82, clone MEL-14, eBioscience, dilution ratio 1:80;
21) Anti-CD4, 17-0041-83, clone GK1.5, eBioscience, dilution ratio 1:150;
22) Anti-CD4, 15-0041-83, clone GK1.5, eBioscience, dilution ratio 1:300;
23) Anti-Foxp3, 12-5773-82, clone FJK-16s, eBioscience, dilution ratio 1:20;
24) Anti-CD45, 17-0451-82, clone 30-F11, eBioscience, dilution ratio 1:150;
25) Anti-CD45, 45-0451-82, clone 30-F11, eBioscience, dilution ratio 1:150;
26) Anti-NFAT1, MA1-025, clone 25A10.D6.D2, Invitrogen, dilution ratio 1:1000;
27) Anti-PCNA, ARG62605, clone PC10, Arigo biolaboratories, dilution ratio 1:1000;
28) Anti-Cleaved caspase-3 (CC3), 9661, clone Asp175, Cell signaling technology, dilution ratio 1:500;
29) Anti-GFP, sc-9996, clone B-2, Santa cruz biotechnology, dilution ratio 1:200;
30) Anti-β-actin, 3700, clone 8H10D10, Cell signaling technology, dilution ratio 1:2000;
31) Anti-CD28, 553295, clone 37.51, BD Pharmingen, dilution ratio 1:200;
32) Anti-CD25, 20-0251-U100, clone PC61.5, Tonbo Bioscience, dilution ratio 1:600;
33) Anti-CD69, 12-0691-82, clone H1.2F3, eBioscience, dilution ratio 1:350;
34) Anti-CD107a, 121614, clone 1D4B, BioLegend, dilution ratio 1:20;
35) Anti-Phospho-FAK (Tyr397), 700255, clone 31H5L17, Invitrogen, dilution ratio 1:200;
36) Anti-Phospho-ERK1/2 (Thr202/Tyr204), 369504, clone 6B8B69, BioLegend, dilution ratio 1:20.

Validation

1) Anti-PD-1, P372, clone RMP1-14, Leinco,
validated for in vivo application and tested to detect in mouse by the manufacturer (https://www.leinco.com/p/anti-mouse-pd-1-cd279-purified-functional-grade-platinum/);

2) Anti-PD-1, 11-9985-82, clone J43, eBioscience,
validated for flow cytometry and tested to detect in mouse by the manufacturer (https://www.thermofisher.com/antibody/product/CD279-PD-1-Antibody-clone-J43-Monoclonal/11-9985-82);

3) Anti-PD-1, 17-9981-82, clone RMP1-30, eBioscience,
validated for flow cytometry and tested to detect in mouse by the manufacturer (https://www.thermofisher.com/antibody/product/CD279-PD-1-Antibody-clone-RMP1-30-Monoclonal/17-9981-82);

4) Anti-PD-1, 12-9985-82, clone J43, eBioscience,
validated for flow cytometry and tested to detect in mouse by the manufacturer (https://www.thermofisher.com/antibody/product/CD279-PD-1-Antibody-clone-J43-Monoclonal/12-9985-82);

5) Anti-PD-1, ab214421, clone EPR20665, abcam,
validated for immunohistochemistry (IHC) analysis and tested to detect in mouse by the manufacturer (https://www.abcam.com/products/primary-antibodies/pd1-antibody-epr20665-ab214421.html);

6) Anti-CD16/32, 156604, clone S17011E, BioLegend,
validated for flow cytometry and tested to detect in mouse by the manufacturer (https://www.biolegend.com/en-ie/products/trustain-fcx-plus-anti-mouse-cd16-32-antibody-17085);

7) Anti-CD8, F398-84A-G, clone KT15, Proimmune,
validated for flow cytometry and tested to detect in mouse by the manufacturer (https://www.proimmune.com/introduction-to-pentamers/);

8) Anti-CD8, 45-0081-82, clone 53-6.7, eBioscience,
validated for flow cytometry and tested to detect in mouse by the manufacturer (https://www.thermofisher.com/antibody/product/CD8a-Antibody-clone-53-6-7-Monoclonal/45-0081-82);

9) Anti-CD8, 12-0081-83, clone 53-6.7, eBioscience,
validated for flow cytometry and tested to detect in mouse by the manufacturer (https://www.thermofisher.com/antibody/product/CD8a-Antibody-clone-53-6-7-Monoclonal/12-0081-83);

10) Anti-CD8, 85336, clone D8A8Y, Cell signaling technology,
validated for immunohistochemistry (IHC) analysis and tested to detect in mouse by the manufacturer (https://www.cellsignal.com/products/primary-antibodies/cd8a-d8a8y-rabbit-mab/85336);

11) Anti-CD3, 45-0031-82, clone 145-2C11, eBioscience,
validated for flow cytometry and tested to detect in mouse by the manufacturer (https://www.thermofisher.com/antibody/product/CD3e-Antibody-clone-145-2C11-Monoclonal/45-0031-82);

12) Anti-CD3, 11-0031-85, clone 145-2C11, eBioscience,
validated for flow cytometry and tested to detect in mouse by the manufacturer (https://www.thermofisher.com/antibody/product/CD3e-Antibody-clone-145-2C11-Monoclonal/11-0031-82);

13) Anti-CD3, 100340, clone 145-2C11, Biolegend,
validated for T cell activation assays and tested to detect in mouse (PMID: 17404269);

14) Anti-CD3, 78588, clone E4T1B, Cell signaling technology,
validated for immunohistochemistry (IHC) analysis and tested to detect in mouse by the manufacturer (https://www.cellsignal.com/products/primary-antibodies/cd3e-e4t1b-xp-rabbit-mab/78588);

15) Anti-Ki67, 652404, clone 16A8, BioLegend,
validated for flow cytometry and tested to detect in mouse by the manufacturer (https://www.biolegend.com/nl-be/products/pe-anti-mouse-ki-67-antibody-8134);

16) Anti-Ki67, ab16667, clone SP6, abcam,
validated for immunohistochemistry (IHC) analysis and tested to detect in mouse by the manufacturer (https://www.abcam.com/products/primary-antibodies/ki67-antibody-sp6-ab16667.html);

17) Anti-Granzyme B, 372204, clone QA16A02, BioLegend,
validated for flow cytometry and tested to detect in mouse by the manufacturer (https://www.biolegend.com/en-us/products/apc-anti-human-mouse-granzyme-b-recombinant-antibody-14429?GroupID=GROUP28);

18) Anti-CD44, 12-0441-82, clone IM7, eBioscience,
validated for flow cytometry and tested to detect in mouse by the manufacturer (https://www.thermofisher.com/antibody/product/CD44-Antibody-clone-IM7-Monoclonal/12-0441-82);

19) Anti-CD62L, 17-0621-82, clone MEL-14, eBioscience,
validated for flow cytometry and tested to detect in mouse by the manufacturer (https://www.thermofisher.com/antibody/product/CD62L-L-Selectin-Antibody-clone-MEL-14-Monoclonal/17-0621-82);

20) Anti-CD62L, 25-0621-82, clone MEL-14, eBioscience,
validated for flow cytometry and tested to detect in mouse by the manufacturer (https://www.thermofisher.com/antibody/product/CD62L-L-Selectin-Antibody-clone-MEL-14-Monoclonal/25-0621-82);

21) Anti-CD4, 17-0041-83, clone GK1.5, eBioscience,
validated for flow cytometry and tested to detect in mouse by the manufacturer (https://www.thermofisher.com/antibody/product/CD4-Antibody-clone-GK1-5-Monoclonal/17-0041-83);

22) Anti-CD4, 15-0041-83, clone GK1.5, eBioscience,
validated for flow cytometry and tested to detect in mouse by the manufacturer (https://www.thermofisher.com/antibody/product/CD4-Antibody-clone-GK1-5-Monoclonal/15-0041-83);

23) Anti-Foxp3, 12-5773-82, clone FJK-16s, eBioscience,
validated for flow cytometry and tested to detect in mouse by the manufacturer (https://www.thermofisher.com/antibody/product/FOXP3-Antibody-clone-FJK-16s-Monoclonal/12-5773-82);

24) Anti-CD45, 17-0451-82, clone 30-F11, eBioscience,
validated for flow cytometry and tested to detect in mouse by the manufacturer (https://www.thermofisher.com/antibody/product/CD45-Antibody-clone-30-F11-Monoclonal/17-0451-82);

25) Anti-CD45, 45-0451-82, clone 30-F11, eBioscience,
validated for flow cytometry and tested to detect in mouse by the manufacturer (https://www.thermofisher.com/antibody/product/CD45-Antibody-clone-30-F11-Monoclonal/45-0451-82);

26) Anti-NFAT1, MA1-025, clone 25A10.D6.D2, Invitrogen,
validated for western blot (WB) and tested to detect in mouse by the manufacturer (https://www.thermofisher.com/antibody/product/NFATC2-Antibody-clone-25A10-D6-D2-Monoclonal/MA1-025);

27) Anti-PCNA, ARG62605, clone PC10, Arigo biolaboratories,
validated for western blot (WB) and tested to detect in mouse by the manufacturer (https://www.arigobio.com/anti-PCNA-antibody-PC10-ARG62605.html);

28) Anti-Cleaved caspase-3 (CC3), 9661, clone Asp175, Cell signaling technology,
validated for immunohistochemistry (IHC) analysis and tested to detect in mouse by the manufacturer (https://www.cellsignal.com/products/primary-antibodies/cleaved-caspase-3-asp175-antibody/9661);

29) Anti-GFP, sc-9996, clone B-2, Santa cruz biotechnology,
validated for immunohistochemistry (IHC) analysis and tested to detect in mouse by the manufacturer (https://www.scbt.com/p/gfp-antibody-b-2);

30) Anti-β-actin, 3700, clone 8H10D10, Cell signaling technology,
validated for western blot (WB) and tested to detect in mouse by the manufacturer (https://www.cellsignal.com/products/primary-antibodies/b-actin-8h10d10-mouse-mab/3700);

31) Anti-CD28, 553295, clone 37.51, BD Pharmingen,
validated for T-cell costimulation and tested to detect in mouse by the manufacturer (https://www.bdbiosciences.com/en-us/products/reagents/flow-cytometry-reagents/research-reagents/single-color-antibodies-ruo/purified-hamster-anti-mouse-cd28.553295);

32) Anti-CD25, 20-0251-U100, clone PC61.5, Tonbo Bioscience,
validated for flow cytometry and tested to detect in mouse by the manufacturer (https://cytekbio.com/products/apc-anti-mouse-cd25-pc61-5?variant=40581238128676);

33) Anti-CD69, 12-0691-82, clone H1.2F3, eBioscience,
validated for flow cytometry and tested to detect in mouse by the manufacturer (https://www.thermofisher.com/antibody/product/CD69-Antibody-clone-H1-2F3-Monoclonal/12-0691-82);

34) Anti-CD107a, 121614, clone 1D4B, BioLegend,
validated for flow cytometry and tested to detect in mouse by the manufacturer (https://www.biolegend.com/de-at/products/apc-anti-mouse-cd107a-lamp-1-antibody-6081);

35) Anti-Phospho-FAK (Tyr397), 700255, clone 31H5L17, Invitrogen,
validated for flow cytometry and tested to detect in mouse by the manufacturer (https://www.thermofisher.com/antibody/product/Phospho-FAK-Tyr397-Antibody-clone-31H5L17-Recombinant-Monoclonal/700255);

36) Anti-Phospho-ERK1/2 (Thr202/Tyr204), 369504, clone 6B8B69, BioLegend,
validated for flow cytometry and tested to detect in mouse by the manufacturer (https://www.biolegend.com/nl-nl/products/alexa-fluor-647-anti-erk1-2-phospho-thr202-tyr204-antibody-12924).

Eukaryotic cell lines

Policy information about cell lines and Sex and Gender in Research

Cell line source(s)	RAW264.7, 4T1, EMT6, and B16F10 cell lines were purchased from ATCC. 4T1-GFP-luc and EMT6-GFP-luc cell lines were then generated by Dr. Kai Miao at University of Macau. CD8+ T cells were isolated from the spleen of BALB/c female mouse.
Authentication	The parental RAW264.7, 4T1, EMT6, and B16F10 cell lines were authenticated by the supplier (ATCC). CD8+ T cells were isolated by commercial EasySep™ Mouse CD8+ T cell isolation kit (catalog No. 19853; Stemcell).
Mycoplasma contamination	All cell lines in this study get tested without mycoplasma contamination.

Commonly misidentified lines (See ICLAC register)	No commonly misidentified cell lines were used.

Animals and other research organisms

Policy information about studies involving animals; ARRIVE guidelines recommended for reporting animal research, and Sex and Gender in Research

Laboratory animals	Female BALB/c mice with the age of 8 weeks were supplied by the Animal Center in the Faculty of Health Sciences, University of Macau. Animals were bred and housed with the light cycle of 12:12, ambient temperature at 22 degree Celsius, and relative humidity range between 40-70%.
Wild animals	No wild animal was involved in this study.
Reporting on sex	Female BALB/c mice were used in the study.
Field-collected samples	No field-collected sample was included in this study.
Ethics oversight	All mouse studies were carried out following the protocols (protocol ID: UMARE-030-2021 and UMAEC-015-2019) approved by the Animal Ethics Committee in University of Macau.

Note that full information on the approval of the study protocol must also be provided in the manuscript.

Flow Cytometry

Plots

Confirm that:

- [x] The axis labels state the marker and fluorochrome used (e.g. CD4-FITC).
- [x] The axis scales are clearly visible. Include numbers along axes only for bottom left plot of group (a 'group' is an analysis of identical markers).
- [x] All plots are contour plots with outliers or pseudocolor plots.
- [x] A numerical value for number of cells or percentage (with statistics) is provided.

Methodology

Sample preparation	Tissues including primary solid tumor, postsurgical wound debris, SLN, and spleen were harvested for single-cell dissociation. Briefly, except for the spleen that avoided the digestion step, other collected tissues were mechanically minced with a sterilized scalpel and then digested in DMEM/F-12 medium, containing 300 U/ml of collagenase type III, 100 U/ml of hyaluronidase, 5% (v/v) FBS, 5 µg/ml of insulin, 10 ng/ml of epidermal growth factor protein, and 500 ng/ml of hydrocortisone for one hour, at 37 °C and 5% (v/v) carbon dioxide. The resultant suspension was then dispersed in DMEM/F-12 medium with 5 mg/ml of dispase II and 0.1 mg/ml of DNase I for 5 min at 37 °C. Red blood cell lysis buffer was followed to remove red blood cells within 3 min. The 70-µm cell strainer was used in the final step to collect single-cell suspension. LIVE/DEAD™ fixable near-IR dead cell stain kit was applied first to differentiate live and dead cells. Cells were then blocked with 0.25 µg anti-mouse CD16/32 antibodies (aCD16/32) per million cells for 10 min on ice prior to immunostaining.
Instrument	CytoFLEX S flow cytometer (Beckman)
Software	FlowJo software (v10.0.7)
Cell population abundance	At least 10,000 cells were analyzed per condition.
Gating strategy	Cells was first gated on FSC-A and SSC-A. Single cells were usually gated using SSC-H and SSC-A. Surface antigen gating was performed on the live cell population.

- [x] Tick this box to confirm that a figure exemplifying the gating strategy is provided in the Supplementary Information.
